# Performance Characteristics of Automobile Air Conditioning Using the R134a/R1234yf Mixture

**DOI:** 10.3390/e22010004

**Published:** 2019-12-19

**Authors:** Yunchan Shin, Taejung Kim, Areum Lee, Honghyun Cho

**Affiliations:** 1 Department of Mechanical Engineering, Graduate School of Chosun University, Gwangju 61452, Korea; sinyunchan@naver.com (Y.S.); leear@chosun.kr (A.L.); 2 Department of Automobile, Yeongju Campus of Korea Polytechnics VI, Yeongju 36142, Korea; 3 Department of Mechanical Engineering, Chosun University, Gwangju 61452, Korea

**Keywords:** COP (coefficient of performance), exergy destruction, exergy efficiency, GWP (global warming potential), R1234yf

## Abstract

In this study, the energy and exergy of an automobile refrigeration system using R134a and R134a/R1234yf were analyzed experimentally with respect to outdoor air temperature and compressor speed. As outdoor air temperature increased from 32.5 °C to 37.5 °C, the coefficient of performance (COP) and total exergy destruction rate of the refrigeration system using Mix30 decreased by 5.19% and 25.8% on average, compared to that of the system using R134a. The exergy efficiency of the Mix30 refrigeration system was on average 21.8% higher than that of the R134a system. As the compressor rotating speed increased from 1000 to 2000 rpm, the cooling capacity of the refrigeration system using R134a and R134a/R1234yf increased, while the COP decreased. The COP and total exergy destruction rate of the refrigeration system using Mix30 decreased by 4.82% and 19.5%, compared to that of the system using R134a. The exergy efficiency of the Mix30 refrigeration system increased on average by 20.7%, compared to that of the R134a system. The total exergy destruction rate of the automobile refrigeration system using R134a/R1234yf decreased with increase in R1234yf, while exergy efficiency increased. In addition, the exergy destruction rate of the automobile refrigeration system decreased as the amount of R1234yf in the R134a/R1234yf automobile refrigeration system increased.

## 1. Introduction

The hydrochlorofluorocarbon (HCFC) refrigerant, which is an alternative to the CFC refrigerant, is generally used in the refrigeration industry. R22 is a representative HCFC refrigerant. Use of the R22 refrigerant is regulated globally because it destroys the ozone layer and causes global warming. R134a is another representative HCFC refrigerant, which is widely used in automotive air-conditioning systems. R134a has advantages, such as excellent thermal stability, non-corrosiveness, and low toxicity. Moreover, its ozone depletion potential (ODP) is zero. However, the global warming potential (GWP) of R134a is 1430, which is very high. The European Commission regulates the use of refrigerants above 150 GWP in air-conditioning systems of cars manufactured after 2017. However, the refrigerant used in the air-conditioning systems of most automobiles currently on the road is still R134a. As a result, environmental problems, including global warming, continue to increase. 

Studies on the application and development of alternative refrigerants for automobiles are ongoing across the globe. Lee at al. [1] evaluated the performance of a freezer/refrigerator employing R1234yf, which is an alternative to R134a, for domestic use. The results revealed that the optimum amount of refrigerant was similar to that of R134a and that the two refrigerants exhibited similar characteristics with regard to power consumption and system performance at a high temperature (43 °C). Some researchers have theoretically compared the thermodynamic properties of R1234yf and R1234ze(E) refrigerants. The results confirmed that performance similar to that of the R134a system can be maintained through simple design changes, e.g., using a suction-line and liquid-line heat exchanger (SLHX) and changing refrigerant circuits [2,3,4]. Other researchers [5,6,7] carried out research to recover waste heat by using the organic rankine cycle (ORC) system in diesel engines to improve efficiency. Yuan et al. [8] calculated CO_2_-converted emissions in China when R134a, R152a, R1234yf, and R744 were used in a mobile air conditioning (MAC) system, according to the amount of refrigerant discharged during operation. Pérez-García et al. [9] carried out a study on second law analysis of a mobile air conditioning system (MACs) using an internal heat exchanger (IHX) with R134a, R1234yf, and R1234ze. Bolaji et al. [10] compared the average performance coefficient and pull-down time for a refrigerator using R152a and R32. They reported that the R152a refrigerant is a viable alternative to R134a because its use can significantly reduce environmental effects. Additionally, Zhao [11] investigated the performance parameters of R1234yf and R744 and conducted various experiments on performance of the refrigeration system. They reported that R744 had excellent operability under low-temperature conditions. Murat et al. [12] developed an experimental automobile air-conditioning system using R1234yf as a refrigerant. A support vector regression (SVR) model of the automobile air conditioner was developed experimentally via evaluation of various performance parameters under a wide range of operating conditions. They reported that the SVR model had good prediction performance, with a mean relative error of 2.01–3.00%, compared to the experimental results. Jignesh et al. [13] theoretically evaluated the performance of various refrigerants (R290, R600a, R407C, R410A, R404A, R152a, and R1234yf) as alternatives to R134a. They found that the most suitable alternative was R1234yf, from a thermodynamic viewpoint. El-Sayed et al. [14] reviewed previous research on environment friendly refrigerants to substitute HCFC and HFCs. Pottker et al. [15] investigated the effect of supercooling of the condenser on the performance of a refrigeration system using R134a and R1234yf under the same operating conditions. They reported that the COP of the R1234yf and R134a system increased by 9% and 18%, respectively.

Comparative studies were performed on the performance and exergy of R134a and R1234yf refrigeration systems, revealing that the cooling capacity and COP of the R1234yf refrigeration system were 3.0–7.0% and 3.6–4.5% lower, respectively, than those of the R134a refrigeration system [16,17,18,19,20,21]. Devecioğlu et al. [22] analyzed the performance of a mobile air-conditioning system using R1234yf, R444A, and R445A (which have a low GWP) and found that the cooling capacity of the system using R444A and R445A was higher than that of the system using R1234yf. However, the COP of the system using R444A and R445A was lower than that of the system using R1234yf, owing to the increase in power consumption of the compressor. Yu et al. [23] analyzed the performance of a CO_2_ automotive air-conditioning system using a mixture of CO_2_ and propane. They reported that the COP of the CO_2_/propane mixture system with a CO_2_ mass fraction of 60% was 29.4% higher than that of the CO_2_ system and similar to that of an R134a system, at the same compressor speed. Golzari et al. [24] analyzed the exergy efficiency to investigate the potential of R1234yf as an alternative to R134a in automotive air-conditioning systems. They found that the use of R1234yf increased exergy efficiency, compared to the R134a system, and maximum entropy generation and exergy destruction occurred in the compressor.

Various mixed refrigerants have been studied in the literature. Zhong et al. [25] measured the specific heat capacity (*c_v_*) and isothermicity of an R1234yf/R290 binary mixture using an adiabatic calorimeter. Dang et al. [26] measured the viscosity of R1234yf, R32/R1234yf, and R125/R1234yf in a single-phase liquid state. The mean absolute deviations of the liquid density of the R32/R1234yf and R125/R1234yf mixtures were 2.8% and 1.3%, respectively, according to the Grunberg–Nissan method. The mean absolute deviations of the liquid-phase density of the R32/R1234yf and R125/R1234yf mixtures according to REFPROP V9.1 [27] were 3.5% and 2.4%, respectively. Meng et al. [28] investigated the performance of an automotive air-conditioning system with a microchannel heat exchanger using an R134a/R1234yf mixture with a ratio of 89:11. Experimental results showed that the capacity of air conditioning using R1234yf/R134a was similar to that using R134a in the cooling and heating modes. However, the COP of the R1234yf/R134a refrigeration system decreased by 4–9% and 4–16% in the cooling and heating modes, respectively, compared to that of the R134a system. Yang et al. [29] conducted experiments using R134a and R1234yf/R134a (R513A, 44/66 wt%) in a domestic refrigerator. It was confirmed that the optimum charge amount of the R513A system was 5.9% less than that of the R134a system, and the pull-down time of the R513A system was reduced by 21%, compared to the R134a system. Aprea et al. [30,31] investigated a method to replace R134a with an HFO refrigerant (R1234yf, R1234ze(E)) and its binary mixture in a domestic refrigerator. Experiments were performed using R1234yf, R1234ze(E), R1234yf/R134a (90/10 wt%), and R1234ze(E)/R134a (90/10 wt%) mixtures. The results showed that the GWP was reduced for both mixtures and that the lifecycle climate performance index of the refrigerator using the R134a/R1234yf mixture decreased by 17%, compared to that of the refrigerator using R134a. Lee et al. [32] experimentally compared the performance of a heat pump using R134a/R1234yf (10/90 wt%) and R134a under summer and winter conditions and reported that the COP and capacity of the heat pump using R134a/R1234yf were similar to those of the heat pump using R134a. Yataganbaba et al. [33] theoretically analyzed the exergy of a vapor compression refrigeration system with two evaporators when R1234yf and R1234ze(E) were used and found that exergy efficiency was significantly influenced by temperature changes in the evaporator and condenser. Hesse [34] investigated the performance of a secondary refrigerant system using R744 in a vehicle air conditioning system with R1234yf and R290.

Generally, the alternative refrigerants to R134a investigated in previous studies were R1234yf and R1234ze(E). Table 1 presents the thermodynamic properties of R134a, R1234yf, and the R134a/R1234yf mixture. The GWP of R134a and R1234yf is 1430 and 4, respectively. The GWP of the R134a/R1234yf mixture decreases with increase in R1234yf content; therefore, a considerable amount of R1234yf must be included in the R134a/R1234yf mixture to satisfy the regulation of the European Union. R1234yf is classified as an alternative refrigerant in the automobile refrigeration system and has a very low GWP (4) and thermodynamic properties similar to R134a. Moreover, it has excellent compatibility with existing oils; hence, R1234yf can be directly applied to automobiles currently in operation. However, because R1234yf has a lower latent heat of evaporation than R134a, it has a disadvantage—decrease in cooling capacity and COP of the refrigeration system. To overcome this problem, several studies have been performed using an SLHX in an R1234yf refrigeration system; however, this increases the weight of the system and the unit price of the automobile.

As another way to solve this problem, it is necessary to study the application of a mixed refrigerant to an automotive air-conditioning system. Additionally, it is necessary to study performance characteristics when R1234yf is mixed with R134a as a refrigerant for automobiles, because R1234yf has great potential for use in automobile air-conditioning systems as a mixed refrigerant with R134a, which is already used in automobiles. However, studies on the performance characteristics of refrigeration systems using mixed refrigerants have mostly focused on domestic refrigerators. The automobile air-conditioning system has quite different operating conditions because the type of expansion valve is different and an automobile air conditioner has several variables that affect system performance, such as solar radiation effect, ventilating effect of cooled air, complicated space, etc. Even though R1234yf has high applicability in an automobile air-conditioning system, research on applying a mixed R134a/R1234yf refrigerant in the current automobile air-conditioning system is insufficient. In particular, research on the energy and exergy characteristics of an automobile air conditioner with regards to the mixing ratios of R134a and R1234yf is hardly found in the literature. Besides, Europe has banned the use of R134a (GWP > 150 refrigerants) on new vehicles since 2011 and the use of refrigerants above GWP 150 has been prohibited for all vehicles since 2017 [35]. In other countries like South Korea, the R1234yf refrigerant has been used in automobiles manufactured post 2017 [36].

However, many conventional automotive HVAC systems still use R134a. When the R134a refrigerant in existing vehicles is replaced with the R1234yf, which has a GWP of 4, the cost burden is considerable, because unit cost of the R1234yf is 20 times higher than that of R134a [37]. Therefore, it is important to investigate the performance characteristics of an HVAC system with an R134a and R1234yf mixed refrigerant in order to reduce the cost burden and maintain high performance. In order to solve this problem, a recommended method is to mix the two refrigerants. This can greatly decrease GWP and CO_2_-eq/kg, as shown in Table 1. However, significantly increasing the mixing ratio of R1234yf can reduce the performance of the automotive air conditioning system; thus, it is important to determine an optimal mixing ratio and investigate system performance characteristics.

In this study, a mixed R134a/R1234yf refrigerant was applied to an automobile refrigeration system, and a performance test was conducted. The performance test used four types of refrigerants under various operating conditions. The four refrigerants were R134a, Mix10 (R134a (90%)/R1234yf (10%)), Mix20 (R134a (80%)/R1234yf (20%)), and Mix30 (R134a (70%)/R1234yf (30%)). Additionally, according to the experimental results, the performance and exergy of the automobile refrigeration system were analyzed. This study provides basic data for optimum design (performance improvement and operating characteristics) of the automobile refrigeration system.

## 2. Experiment and Methods 

**Figure 1:**
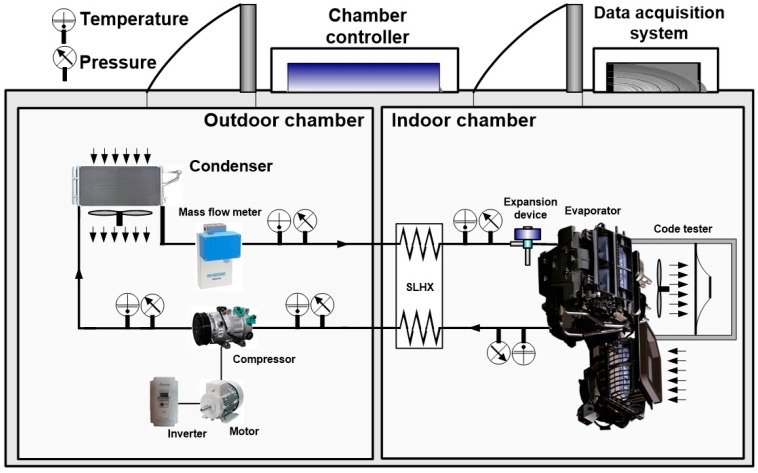
Schematic diagram of the experimental setup.

**Figure 2:**
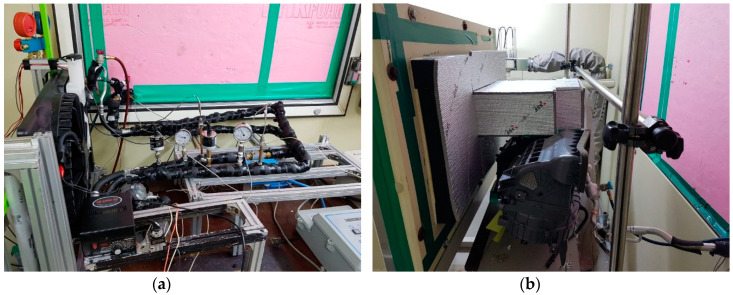
Photographs of the experimental setup: (**a**) Outdoor; (**b**) Indoor.

The automobile air conditioner used in this study consisted of a compressor, a condenser, an evaporator, an expansion device (TXV), and an SLHX. Figure 1, Figure 2 show a schematic diagram and photographs, respectively, of the experimental setup. Table 2 presents detailed specifications of the components of an automobile refrigeration system. A reciprocating compressor was used in the experiment, which was connected to an electric motor equipped with an inverter to control the rotating speed of the compressor. 

A fin-tube-type heat exchanger with a louver fin was used as a condenser and evaporator, and the TXV (thermostatic expansion valve) was installed as an expansion device in the automobile refrigeration system. The SLHX was a double-tube heat exchanger with inner and outer tube diameters of 9 mm and 19 mm, and length of 38 cm. T-type thermocouples and pressure gauges were installed between the components to measure the thermodynamic properties of the main parts of the system. A mass flow meter was installed at the outlet of the compressor to measure the mass flow rate of the refrigerant in the system. Additionally, a power meter was used to measure power consumption in the compressor, and refrigerant mass charge in the system was measured using a mass scale. Table 3 presents the detailed specifications and accuracy of the measuring devices used in this study. The uncertainties of compressor work and cooling capacity of the system was 2.26% and 4.72%, respectively. Besides, total uncertainty of the system, COP, was 4.99%.

To evaluate the performance of the automobile refrigeration system, R134a was used and its optimum charge amount determined. Under optimal charge conditions, performance of the R134a system was investigated according to the operating conditions. Subsequently, the performance of the R134a/R1234yf system was estimated. In this study, the mass mixing ratios of R134a/R1234yf were set at 90:10, 80:20, and 70:30, taking into consideration optimum charge amounts of the R134a system. Table 4 presents the experimental conditions. The performance test of the automobile refrigeration system was conducted with respect to outdoor air temperature and compressor rotating speed.

Using the performance test results of the automobile refrigeration system, performance characteristics of the system, taking into consideration operating conditions, were analyzed. Additionally, the exergy destruction rates of the components in the system were calculated and compared by using the obtained property date. Generally, the heat-exchange rate at the SLHX is very small, compared to that of the other components, and its variation with operating conditions is miniscule. Therefore, energy and exergy analysis was performed for the main components (compressor, condenser, expansion value, and evaporator). In addition, the total exergy destruction rates and exergy efficiencies of the different systems were analyzed and compared. The cooling capacity, COP, exergy destruction rate, total exergy destruction rate, and exergy efficiency of the automobile refrigeration system were calculated using Equations (1)–(8).

(1) $${\dot{Q}}_{evap}=\dot{m}\left(h_{air,in}-h_{air,out}\right)$$

(2) $$COP_{R}=\frac{{\dot{Q}}_{evap}}{{\dot{W}}_{comp}}$$

(3) $$EDR_{comp}=\dot{m}T_{0}\left(S_{comp,out}-S_{comp,in}\right)$$

(4) $$EDR_{cond}=T_{0}\left[\dot{m}\left(S_{cond,out}-S_{cond,in}\right)+\frac{{\dot{Q}}_{cond}}{T_{cond}}\right]$$

(5) $$EDR_{expa}=\dot{m}T_{0}\left(S_{expa,out}-S_{expa,in}\right)$$

(6) $$EDR_{evap}=T_{0}\left[\dot{m}\left(S_{evap,out}-S_{evap,in}\right)-\frac{{\dot{Q}}_{evap}}{T_{evap}}\right]$$

(7) $$Total\;EDR=EDR_{comp}+EDR_{cond}+EDR_{\exp a}+EDR_{evap}$$

(8) $$\eta_{exergy}=\frac{EDR_{evap}}{{\dot{W}}_{comp}}=\frac{{\dot{Q}}_{evap}\left(T_{0}-T_{evap}\right)/T_{evap}}{{\dot{Q}}_{evap}/COP_{R}}$$

## 3. Results

### 3.1. Energy Evaluation of an Automobile Refrigeration System with Respect to Outdoor Air Temperature and Compressor Rotating Speed

**Figure 3:**
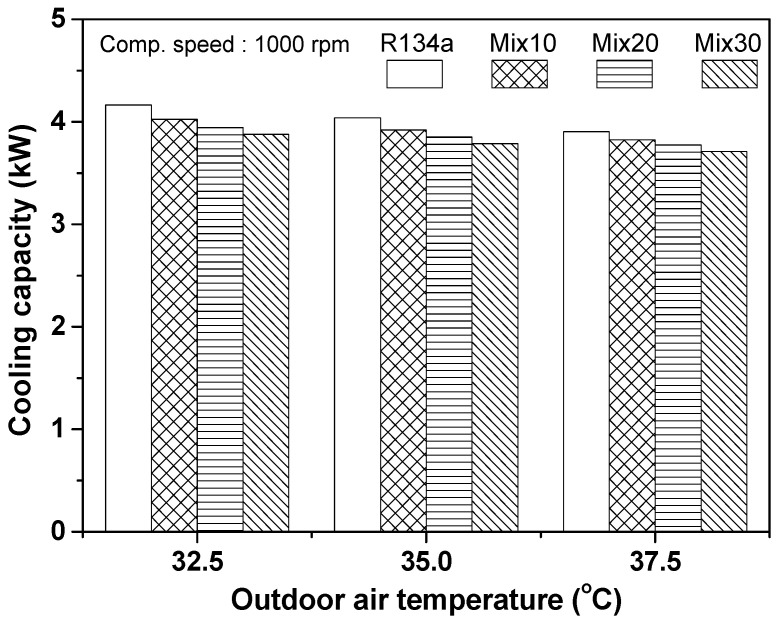
Evaluation of cooling capacity with respect to outdoor air temperature.

Because the performance of the automobile refrigeration system is significantly affected by outdoor air temperature, it is important to investigate system performance taking that aspect into consideration. Figure 3 presents the variation in cooling capacity of the automobile refrigeration system using R134a and the R134a/R1234yf mixture, according to outdoor air temperature. The cooling capacity of the R134a refrigeration system was found to be 4.164 kW when outdoor air temperature was 32.5 °C, and the cooling capacity decreased slightly as the mixing ratio of R1234yf increased in the R134a/R1234yf refrigeration system. In the case of the Mix30 system, which had the highest mixing ratio of R1234yf, the cooling capacity of the system was found to be 3.878 kW, which was 5.85% lower than that of the R134a refrigeration system. At an outdoor air temperature of 35 °C, the cooling capacity of the R134a refrigeration system was 4.039 kW. In the R134a/R1234yf refrigeration system, the cooling capacity of the Mix30 system was 3.787 kW, which was approximately 6.24% lower than that of the R134a system. The cooling capacity of the R134a refrigeration system was 3.904 kW at an outdoor air temperature of 37.5 °C. When the Mix30 was applied to the R134a/R1234yf refrigeration system, the cooling capacity was 3.712 kW, which was approximately 4.92% lower than that of the R134a system. According to Qi et al. [38], the cooling capacity of an R1234yf refrigeration system was approximately 7.7–10.6% lower than that of an R134a system. Their result is consistent with the experimental tendency observed in this study, i.e., the reduction of performance with increase in the R1234yf mixing ratio. In this study, the cooling capacity of the automobile refrigeration system decreased by 4.9–6.9% as the mixing ratio of R1234yf increased in the R134a/R1234yf refrigeration system. This is because the latent heat of evaporation, liquid-phase specific heat, and liquid-phase thermal conductivity of R1234yf are lower than those of R134a. As the outdoor air temperature increased from 32.5 °C to 37.5 °C, the cooling capacity of the automobile refrigeration system decreased. The cooling capacity of the refrigeration system using R134a, Mix10, Mix20, and Mix30 decreased by 6.24%, 5.02%, 4.31%, and 4.28%, respectively. This is because the temperature difference between the refrigerant and the outdoor air in the condenser decreased as the outdoor air temperature increased. Moreover, this effect led to a reduction in evaporator inlet quality. Therefore, the refrigerant effect, i.e., the enthalpy difference of the refrigerant between the evaporator inlet and outlet, decreased. However, the R134a system showed a relatively large reduction in cooling capacity with increase in outdoor air temperature. Moreover, this reduction decreased with increase in the R1234yf mixing ratio in the R134a/R1234yf system. This is because the reduction in latent heat of vaporization and thermal conductivity of the liquid phase of R1234yf with increase in outside air temperature were lesser than those of R134a. Additionally, the increase rates of specific heat and vapor-phase thermal conductivity of R1234yf were higher than that of R134a.

**Figure 4:**
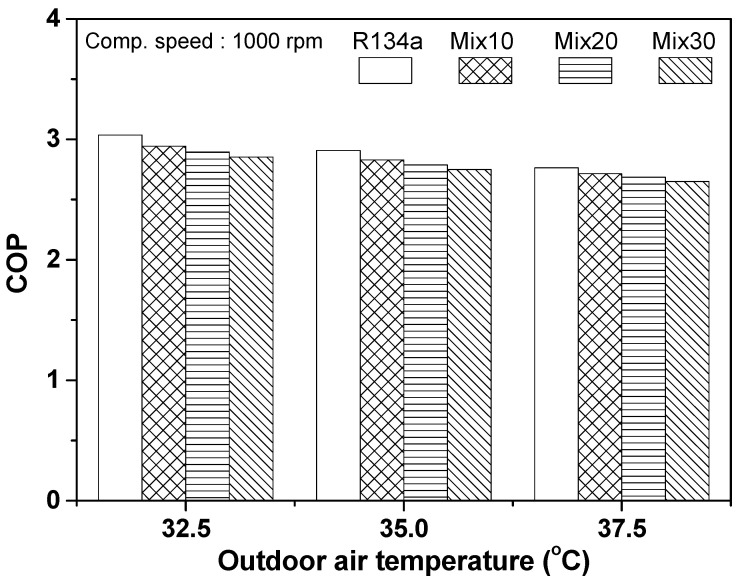
Evaluation of COP with respect to outdoor air temperature.

Figure 4 shows the variation in COP of the automobile refrigeration system using R134a and the R134a/R1234yf mixture, according to the mixing ratio of R1234yf under different outdoor air temperatures. The COP of the R134a refrigeration system was 3.04 at outdoor air temperature of 32.5 °C. The COP of the automobile refrigeration system decreased as the mixing ratio of R1234yf in the R134a/R1234yf refrigeration system increased. In the case of Mix30, which had the largest portion of R1234yf, the COP of the system was 2.854, which was approximately 6.12% lower than that of the R134a system. The COP of the R134a system was 2.91 at an outdoor air temperature of 35 °C. The COP of the Mix30 system was 2.75, which was approximately 5.42% lower than that of the R134a system. When the outdoor air temperature was 37.5 °C, the COPs of the R134a and Mix30 systems were 2.77 and 2.65, respectively; i.e., that of the Mix30 system was approximately 4.12% lower than that of the R134a system. As the outdoor air temperature increased from 32.5 °C to 37.5 °C, the COP of the refrigeration system tended to decrease, and the COP of the system using R134a, Mix10, Mix20, and Mix30 decreased by 8.97%, 7.78%, 7.17%, and 7.08%, respectively. As the outdoor air temperature increased, the COP of the system decreased gradually, and the reduction in performance with increase in outdoor air temperature decreased as the mixing ratio of R1234yf increased. Generally, the performance of refrigeration systems degrades with increase in outdoor air temperature because of the reduction in cooling capacity and increase in compressor power consumption. As the outdoor temperature increases, the cooling capacity decreases, owing to a decrease in cooling effect in the evaporator, because the temperature at the outlet of the condenser increases. Additionally, the compressor power consumption increases due to a rise in compression ratio during the compression process. This reduces the mass flow rate of the refrigerant in the refrigeration system. 

**Figure 5:**
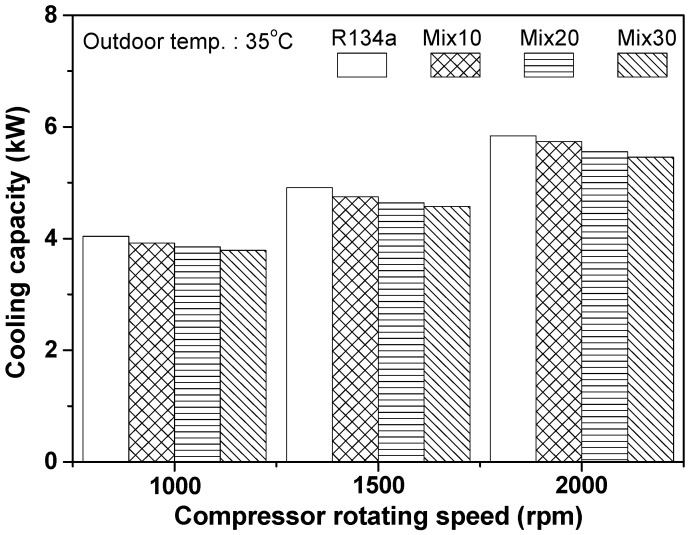
Evaluation of cooling capacity with respect to compressor rotating speed.

Generally, in the case of an automobile refrigeration system, the compressor rotating speed continuously changes from 700 rpm to 3000 rpm, as per driving conditions. To investigate performance variation of an automobile refrigeration system with respect to compressor rotating speed, the cooling capacity of the refrigeration system using R134a and the R134a/R1234yf mixture was measured, as shown in Figure 5. The cooling capacities of the R134a and Mix30 systems were found to be 4.039 and 3.787 kW, respectively, when compressor rotating speed was 1000 rpm; this increased to 5.839 and 5.455 kW, respectively, at a rotating speed of 2000 rpm. The cooling capacity of the Mix30 system was approximately 6.58% lower than that of the R134a system. As the compressor rotating speed increased from 1000 to 2000 rpm, the cooling capacity of the R134a, Mix10, Mix20, and Mix30 refrigeration systems increased by 44.6%, 46.3%, 44.3%, and 44.0%, respectively. In a previous study on the performance of an existing automobile refrigeration system [28], the cooling capacity increased by approximately 23.0% when the compressor rotating speed increased from 2000 to 3000 rpm. The increase in cooling capacity in that study was slightly smaller than the values in this study. This is mainly due to the difference in compressor rotating speed. As the compressor rotating speed increases, the cooling capacity shows an initial rapid increases, but gradually slows down. Therefore, the change in cooling capacity in the previous study is considered to be similar to the result obtained in this study. The mass flow rate in the system increased as the compressor rotating speed increased, resulting in a rise in cooling capacity. However, the increment of the cooling capacity decreased as the compressor rotating speed increased. Additionally, as the mixing ratio of R1234yf in the R134a/R1234yf system increased, the cooling capacity decreased, because the latent heat of evaporation and the liquid-state thermal conductivity of R1234yf were lower than that of R134a. However, the increment in cooling capacity for the R134a/R1234yf refrigeration system, taking into consideration compressor rotating speed, was similar to that of the R134a system.

**Figure 6:**
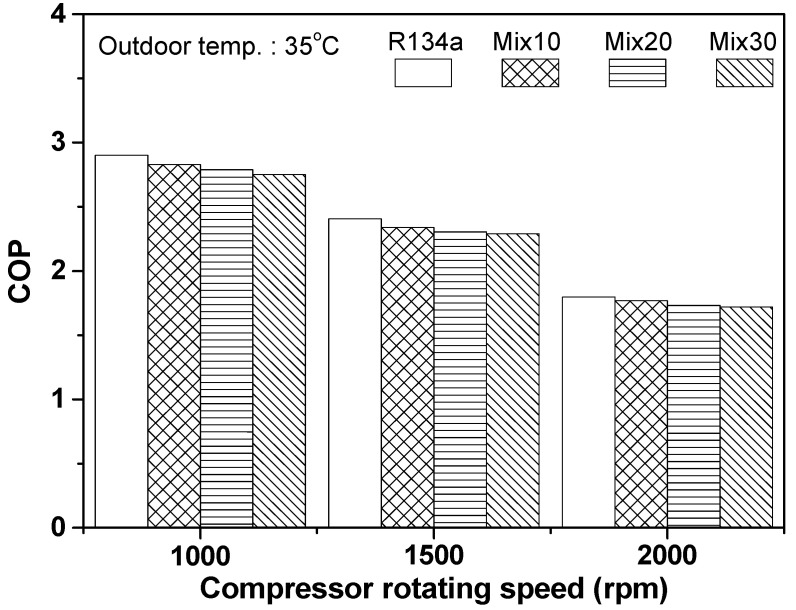
Evaluation of COP with respect to compressor rotating speed.

Figure 6 shows the variation in COP of the automobile refrigeration system using R134a and the R134a/R1234yf mixture, as per compressor rotating speed. At a compressor speed of 1000 rpm, the COPs of the refrigeration systems using R134a and Mix30 were 2.91 and 2.75, respectively; i.e., the COP of the Mix30 system was approximately 5.42% lower than that of the R134a system. The COPs of the R134a and Mix30 systems were 1.80 and 1.72, respectively, at compressor speed of 2000 rpm, and the COP of the Mix30 system was 4.22% lower than that of the R134a system. When the compressor rotating speed increased from 1000 to 2000 rpm, the COP of the refrigeration system decreased. The decrease in the COP for the R134a, Mix10, Mix20, and Mix30 systems was 38.2%, 37.5%, 37.9%, and 37.4%, respectively. The decrease in COP for the R134a/R1234yf system was slightly smaller than that for the R134a system, but the difference was very small. Generally, as the compressor rotating speed increases, both the compressor power consumption and the cooling capacity simultaneously increase, owing to a rise in compression ratio and mass flow rate. However, the COP of the refrigeration system decreases, because the increase in compressor power consumption outweighs the increase in cooling capacity. In a study by Meng et al. [28], the COP of an automobile air conditioner decreased by approximately 17.4% (from 3.8 to 3.14) as the compressor rotating speed increased from 2000 to 3000 rpm. Their experimental results exhibit the same trend as the results of the present study, i.e., the decreasing trend of COP with increase in the compressor rotating speed; however, their decrement in COP was significantly smaller than that in the present study. This is because the experimental conditions, such as outdoor air temperature, air flow rate, and compressor rotation, differed.

### 3.2. Exergy Evaluation of Automobile Refrigeration System with Respect to Outdoor Air Temperature and Compressor Rotating Speed

**Figure 7:**
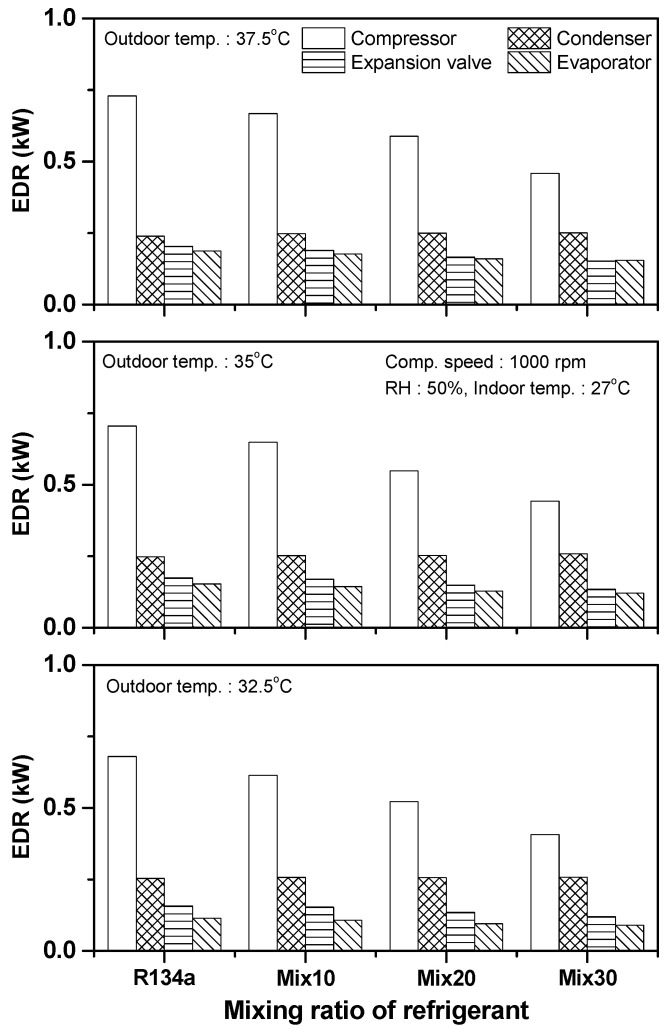
Variation in exergy destruction rate for each component with respect to outdoor air temperature.

Under the criterion that internal heat and work are equivalent, according to the first law of thermodynamics, it is impossible to evaluate the potential performance and efficiency of a thermal system by using energy and mass conservation. Exergy analysis of each component in a thermal system should be performed to investigate the sources of energy loss in the system and to evaluate energy effectiveness. Moreover, it is possible to identify methods for performance improvement using the exergy destruction rate and efficiency of each component. Figure 7 shows the exergy destruction rate of each component according to a mixing ratio of the R134a/R1234yf refrigeration system at various outdoor air temperatures. The exergy destruction rates of the compressor, condenser, expansion device, and evaporator in the R134a system were 0.680, 0.253, 0.157, and 0.114 kW, respectively, at outdoor air temperature of 32.5 °C. When Mix30 was applied to the R134a/R1234yf system, the exergy destruction rates of the compressor, expansion device, and evaporator were reduced by 40.1%, 23.8%, and 21.3%, respectively, compared to that of the R134a system. However, exergy destruction in the condenser increased by 1.54%. The exergy destruction rates of the compressor, condenser, expansion device, and evaporator in the R134a system were 0.705, 0.248, 0.174, and 0.153 kW, respectively, at outdoor air temperature of 35 °C. The exergy destruction rates of the compressor, expansion device, and evaporator in the Mix30 system decreased by 37.3%, 22.6%, and 20.5%, respectively, compared to that of the R134a system, while the exergy destruction rate of the condenser increased by 4.15%. At outdoor air temperature of 37.5 °C, the exergy destruction rates of the compressor, condenser, expansion device, and evaporator in the R134a system were 0.730, 0.239, 0.190, and 0.188 kW, respectively. The exergy destruction rates of the compressor, expansion device, and evaporator in the Mix30 system decreased by 37.2%, 19.7%, and 17.8%, respectively, compared to that of the R134a system, while the exergy destruction rate of the condenser increased by 4.90%. With the increase in the R1234yf ratio, the exergy destruction rates of the compressor, expansion device, and evaporator in the R134a/R1234yf refrigeration system decreased, but the exergy destruction rate of the condenser increased slightly. Additionally, as the outdoor air temperature increased from 32.5 °C to 37.5 °C, the exergy destruction rates of the compressor in the R134a, Mix10, Mix20, and Mix30 systems increased by 7.36%, 8.65%, 12.8%, and 12.6%, respectively, while the exergy destruction rate of the condenser decreased by 5.69%, 3.51%, 2.65%, and 2.57%, respectively. This is because the heat-transfer rate in the condenser decreased as the outdoor air temperature increased; hence, entropy generation was reduced. However, because the compression ratio and discharge refrigerant temperature increased in the compressor, the exergy destruction in the compressor increased owing to a rise in heat loss and heat transfer to the surrounding areas. Furthermore, under variable outdoor temperatures, the exergy destruction rate of the expansion device increased and was within 21–27.6%. 

**Figure 8:**
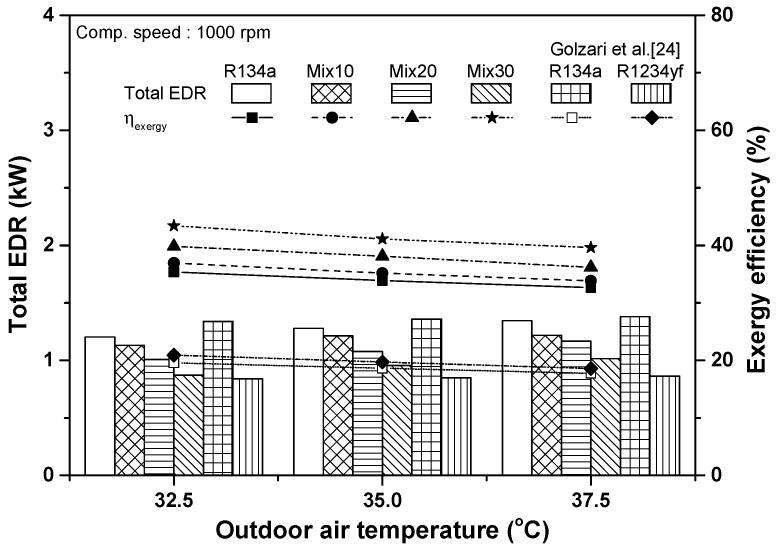
Variations in total exergy destruction rate and exergy efficiency with respect to outdoor air temperature.

Figure 8 shows the variations in total exergy destruction rate and exergy efficiency of the automobile refrigeration system using R134a and the R134a/R1234yf mixture with respect to the outdoor air temperature. The total exergy destruction rates of the R134a system were 1.203, 1.279, and 1.346 kW at outdoor temperatures of 32.5 °C, 35 °C, and 37.5 °C, respectively. Under these conditions, the total exergy destruction rates of the Mix30 system were approximately 27.5%, 25.2%, and 24.6% lower than those of the R134a system. As the ratio of R1234yf in the R134a/R1234yf system increased, the total exergy destruction rate decreased by 25.8% on average at all outdoor temperatures. Our results were compared with those of Golzari et al. [24], who performed an exergy analysis of R134a and R1234yf systems when the inlet air temperature at the condenser was changed from 25 °C to 45 °C under the constant inlet air temperature of the evaporator (27 °C). They reported that the total exergy destruction rate of the R134a and R1234yf systems increased by 3.1% and 2.9%, respectively, when the outdoor air temperature increased from 32.5 °C to 37.5 °C and that the total exergy destruction rate of the R1234yf system was reduced by 37.5% on average, compared to that of the R134a system. These trends match the results of the present study. In this study, when the outdoor temperature increased from 32.5 °C to 37.5 °C, the total exergy destruction rates of the R134a and R134a/R1234yf systems increased by 11.8–16.3%. Additionally, the exergy efficiencies of the systems decreased by 7.8–9.2%. As the ratio of R1234yf increased, the exergy efficiency increased by 21.8% on average at various outdoor temperatures. Golzari et al. [24] also reported that the exergy efficiencies of the R134a and R1234yf systems decreased by 9.3% and 11.2%, respectively, as the outdoor air temperature increased. The decrement of exergy efficiency in the study by Golzari et al. [24] was similar to that in the present study. As the outdoor air temperature increased, the temperature difference between the air and the refrigerant in the condenser decreased, reducing the heat transfer in the condenser. Furthermore, the total exergy destruction rate increased and exergy efficiency decreased because the cooling capacity in the evaporator decreased and the power consumption in the compressor increased.

**Figure 9:**
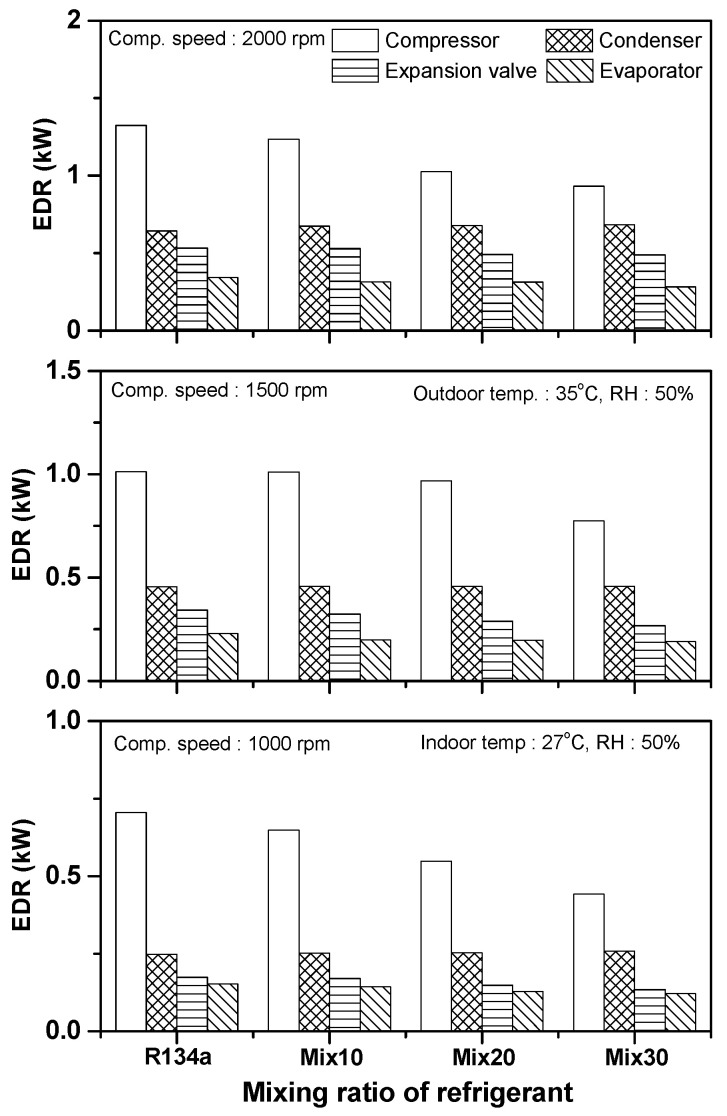
Variation in exergy destruction rate for each component with respect to compressor rotating speed.

Figure 9 shows a comparison of the exergy destruction rates of the different components in the automobile refrigeration system according to the R134a/R1234yf mixing ratio under various compressor rotating speeds. The exergy destruction rates of the compressor, condenser, expansion device, and evaporator in the R134a system were 0.705, 0.248, 0.174, and 0.153 kW, respectively, at a compressor rotating speed of 1000 rpm. The exergy destruction rates of the compressor, expansion device, and evaporator in the Mix30 system decreased by 37.3%, 22.6%, and 20.5%, respectively, compared to that of the R134a system. However, the exergy destruction rate of the condenser increased by 4.15%. The total exergy destruction rate of the R134a system was 3.774 kW when the compressor rotating speed was 1500 rpm, and the exergy destruction rates of the compressor, condenser, expansion device, and evaporator were 1.013, 0.456, 0.343, and 0.230 kW, respectively. When Mix30 was applied to the R134a/R1234yf system, the exergy destruction rates of the compressor, expansion device, and evaporator decreased by 23.6%, 22.0%, and 17.3%, respectively, compared to that of the R134a system, while that of the condenser increased by 0.44%. At the compressor rotating speed of 2000 rpm, the exergy destruction rates of the compressor, condenser, expansion device, and evaporator in the R134a system were 1.323, 0.644, 0.534, and 0.343 kW, respectively. Those of the Mix30 system decreased by 29.5%, 8.5%, and 17.6%, respectively, compared to the R134a system, while the exergy destruction rate of the condenser increased by 6.12%. As the ratio of R1234yf increased in the R134a/R1234yf system, the exergy destruction rates of the compressor, expansion device, and evaporator tended to decrease. As the compressor rotating speed increased, the exergy destruction rates of the compressor, condenser, expansion device, and evaporator in the R134a, Mix10, Mix20, and Mix30 systems increased, resulting in the increase in the total exergy destruction in the systems. With the increase in the compressor rotating speed, the increase in the exergy destruction rate was the largest in the expansion device.

**Figure 10:**
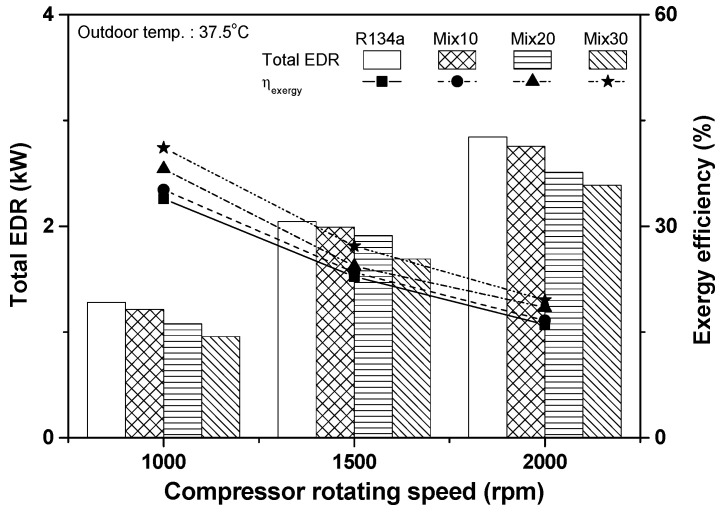
Variations in total exergy destruction rate and exergy efficiency with respect to compressor rotating speed.

Figure 10 shows the variations in total exergy destruction rate and exergy efficiency of the automobile refrigeration systems using R134a and R134a/R1234yf with respect to compressor rotating speed. The total exergy destruction rates of the R134a and Mix30 systems were 1.279 and 0.956 kW, respectively, when the compressor rotating speed was 1000 rpm, and the total exergy destruction rate of the Mix30 system was 25.2% lower than that of the R134a system. At compressor rotating speed of 2000 rpm, the total exergy destruction rates of the R134a and Mix30 systems were 2.843 and 2.386 kW, respectively; i.e., the total exergy destruction rate of the Mix30 system was 16.1% lower than that of the R134a system. The increase in compressor rotating speed increased the total exergy destruction rate of the refrigeration system; however, the addition of R1234yf into the R134a system reduced the total exergy destruction rate. At compressor rotating speed of 1000 rpm, the exergy efficiencies of the R134a and Mix30 systems were 33.9% and 41.1%, respectively; i.e., the exergy efficiency of the Mix30 system was 21.4% higher than that of the R134a system. The exergy efficiencies of the R134a and Mix30 systems at the compressor rotating speed of 2000 rpm were 16.0% and 19.5%, respectively; i.e., the exergy efficiency of the Mix30 system was 21.9% higher than that of the R134a system. As the compressor rotating speed increased from 1000 to 2000 rpm, the exergy efficiencies of the R134a, Mix10, Mix20, and Mix30 systems decreased by 52.7%, 52.7%, 51.5%, and 52.5%, respectively. The exergy efficiency of the R134a/R1234yf system decreased as the mixing ratio of R1234yf increased because the cooling capacity in the evaporator decreased significantly owing to the increase in the R1234yf mixing ratio, but the power consumption in the compressor was not noticeably changed.

The automobile refrigeration system using the R134a/R1234yf mixture can reduce the GWP and generation amount of carbon dioxide according to the mixing ratio of R1234yf. In this study, the performance of an automobile refrigeration system using R134a and the R134a/R1234yf mixture was investigated, according to outdoor air temperature and compressor rotating speed. Additionally, the exergy destruction rates of the main components and the exergy efficiencies of four refrigeration systems were analyzed. As the outdoor air temperature increased from 32.5 °C to 37.5 °C, the cooling capacity and COP decreased, and the average COP of the Mix30 refrigeration system was 5.19% lower than that of the R134a system. Moreover, the total exergy destruction rate of the Mix30 refrigeration system was on average 25.8% lower than that of the R134a system, while the exergy efficiency of the Mix30 refrigeration system was 21.8% higher than that of the R134a system. The COPs of the R134a and R134a/R1234yf refrigeration systems tended to decrease as the compressor rotating speed increased from 1000 to 2000 rpm. The COP of the Mix30 refrigeration system decreased by 4.82% on average, compared to that of the R134a system. Furthermore, on average, the total exergy destruction rate of the Mix30 refrigeration system decreased by 19.5% on average, compared to that of the R134a system; while the exergy efficiency of the Mix30 refrigeration system increased by 20.7%, compared to that of the R134a system. In this study, the COP of the R134a/R1234yf refrigeration system using Mix30 was approximately 4.82% lower than that of the R134a system, but the COP reduction rate was not significant. In addition, the total exergy destruction rate of the Mix30 refrigeration system was lower than that of the R134a system. This indicates that the available energy of the Mix30 refrigeration system was higher than that of the R134a system. It was indirectly confirmed that the R134a/R1234yf mixed refrigerant is applicable to the refrigeration systems of automobiles currently on the road.

## 4. Conclusions

The performance of an automobile refrigeration system using R134a and an R134a/R1234yf mixture was experimentally investigated. As the outdoor air temperature increased from 32.5 °C to 37.5 °C, the COP and cooling capacity of the R134a and R134a/R1234yf refrigeration systems was found to decrease. The COP of the refrigeration systems using R134a and R134/R1234yf decreased by 7.08–8.97%, and the reduction in COP with increase in outdoor temperature decreased as the mixing ratio of R1234yf increased. Moreover, the COP of the Mix30 refrigeration system was 5.19% lower than that of the R134a system, on average. Additionally, as the outdoor air temperature increased from 32.5 °C to 37.5 °C, the exergy destruction rate of the compressor in the R134a and R134a/R1234yf systems increased by 7.36–12.8%, while the exergy destruction rate of the condenser decreased by 2.57–5.69%. Among the main components, the compressor exhibited the highest exergy destruction rate under all operating conditions. The total exergy destruction rate of the Mix30 refrigeration system was averagely 25.8% lower than that of the R134a system, while the exergy efficiency of the Mix30 refrigeration system was 21.8% higher than that of the R134a system. As the compressor rotating speed increased from 1000 to 2000 rpm, the cooling capacity of the R134a and R134a/R1234yf refrigeration systems increased by 44.0–44.6%, while the COP decreased by 37.4–38.2%. The COP of the Mix30 refrigeration system was 4.82% lower than that of the R134a system. The exergy destruction rates of the compressor, condenser, expansion device, and evaporator in the R134a and R134a/1234yf systems tended to increase. The total exergy destruction rate of the Mix30 refrigeration system decreased by 19.5% on average, compared to that of the R134a system, while the exergy efficiency of the Mix30 refrigeration system increased by 20.7%, compared to that of the R134a system.

In this study, the COP of the R134a/R1234yf refrigeration system using Mix30 was approximately 5.01% lower than that of the R134a system. The total exergy destruction rate of the automobile refrigeration system using the R134a/R1234yf mixture decreased with increase in R1234yf. However, the exergy efficiency of the automobile refrigeration system using the R134a/R1234yf mixture increased with increase in R1234yf. Additionally, the total exergy destruction rate of the Mix30 refrigeration system was lower than that of the R134a system, and the available energy of the Mix30 system was higher than that of the R134a system. Therefore, it was indirectly confirmed that the R134a/R1234yf mixed refrigerant is applicable to refrigeration systems of automobiles currently on the road and that a low GWP can be achieved by mixing the two refrigerants appropriately.

## Figures and Tables

**Table 1:** Refrigerant properties of R134a, R1234yf, and R134a/R1234yf.

Property	Unit	R134a	Mix10	Mix20	Mix30	R1234yf
Molecular Weight	-	102.03	103.23	104.43	105.63	114.04
Density Saturated liquid (25 °C) Saturated vapor (25 °C)	kg/m^3^	1206.7 32.35	1194.6 32.87	1182.6 33.39	1170.7 33.93	1091.9 37.93
Specific heat Saturated liquid (25 °C) Saturated vapor (25 °C)	kJ/(kg·K)	1.4246 1.0316	1.4212 1.0349	1.4178 1.038	1.4144 1.0408	1.3921 1.0533
Thermal conductivity Saturated liquid (25 °C) Saturated vapor (25 °C)	mW/(m·K)	81.134 13.825	79.167 13.862	77.247 13.899	75.376 13.934	63.535 13.868
Viscosity Saturated liquid (25 °C) Saturated vapor (25 °C)	μPa·s	194.89 11.693	190.34 11.775	185.9 11.703	181.58 11.634	154.26 11.102
ODP	-	0	0	0	0	0
GWP	-	1430	1287.4	1144.8	1002.2	4
CO_2_-eq/kg	-	1000	900.28	800.56	700.84	2.8

**Table 2:** Specifications of components in the automobile refrigeration system.

Component	Specifications
Condenser	Finned tube type (louver fin), 500 (W) × 400 (H) × 12 (t)
Evaporator	Finned tube type (louver fin), 280 (W) × 200 (H) × 35 (t)
Expansion valve	TXV type (block), 5.3 kW (1.5RT)
Compressor	Reciprocating type (belt-driven), volumetric flow rate: 8.0 m^3^/h
SLHX	Double tube type, D_inner_: 9 mm, D_outer_ = 19 mm, Length = 38 cm

**Table 3:** Specifications of the experimental devices.

Parameter	Type	Range	Accuracy
Temperature	T-type thermocouples	−200–300 °C	±1 °C
Pressure	Setra C206 pressure gauge	−14.7–3000 psig	±0.13%
Mass flow rate	RHM 04	0.05–10 kg/min	0.1%
Power meter	WT230	180–264 VAC	±0.1%
Mass scale	FC-100K	0.02–100 kg	±20 g

**Table 4:** Test conditions.

Item	Conditions
Mixing ratio (R134a:R1234yf)	Mix10(90:10)/Mix20(80:20)/Mix30(70:30)
Compressor speed (rpm)	1000 */1500/2000
Outdoor air temperature (°C)	32.5/35 */37.5
Indoor temperature (°C)	27
Relative humidity (%)	50

* standard condition.

## Nomenclature

CFC	Chlorofluorocarbon
COP	Coefficient of performance
D	Diameter (m)
EDR	Exergy destruction rate (kW)
GWP	Global warming potential
H	Enthalpy (kJ/kg)
HCFC	Hydrochlorofluorocarbon
m	Mass flow rate (kg/s)
ODP	Ozone depletion potential
Q	Cooling capacity (kW)
RH	Relative humidity (%)
s	Entropy (kJ/kg·K)
SLH	Suction-line liquid-line heat exchanger
T	Temperature (K)
TXV	Thermal expansion valve
W	Compressor work (kW)
**Greek Symbol**	
η	efficiency
**Subscripts**	
comp	Compressor
cond	Condenser
expa	Expansion valve
evap	Evaporator
in	Inlet
out	Outlet
0	Dead state
